# Biophysical Characterization of the Olfactomedin Domain of Myocilin, an Extracellular Matrix Protein Implicated in Inherited Forms of Glaucoma

**DOI:** 10.1371/journal.pone.0016347

**Published:** 2011-01-24

**Authors:** Susan D. Orwig, Raquel L. Lieberman

**Affiliations:** School of Chemistry and Biochemistry, Georgia Institute of Technology, Atlanta, Georgia, United States of America; Institut Européen de Chimie et Biologie, France

## Abstract

Myocilin is an eye protein found in the trabecular extracellular matrix (TEM), within the anatomic region that controls fluid flow. Variants of myocilin, localized to its olfactomedin (OLF) domain, have been linked to inherited forms of glaucoma, a disease associated with elevated intraocular pressure. OLF domains have also been implicated in psychiatric diseases and cancers by their involvement in signaling, neuronal growth, and development. However, molecular characterization of OLFs has been hampered by challenges in recombinant expression, a hurdle we have recently overcome for the myocilin OLF domain (myoc-OLF). Here, we report the first detailed solution biophysical characterization of myoc-OLF to gain insight into its structure and function. Myoc-OLF is stable in the presence of glycosaminoglycans, as well as in a wide pH range in buffers with functional groups reminiscent of such glycosaminoglycans. Circular dichroism (CD) reveals significant β-sheet and β-turn secondary structure. Unexpectedly, the CD signature is reminiscent of α-chymotrypsin as well as another ocular protein family, the βγ-crystallins. At neutral pH, intrinsic tryptophan fluorescence and CD melts indicate a highly cooperative transition with a melting temperature of ∼55°C. Limited proteolysis combined with mass spectrometry reveals that the compact core structural domain of OLF consists of approximately residues 238-461, which retains the single disulfide bond and is as stable as the full myoc-OLF construct. The data presented here inform new testable hypotheses for interactions with specific TEM components, and will assist in design of therapeutic agents for myocilin glaucoma.

## Introduction

**Figure 1:**
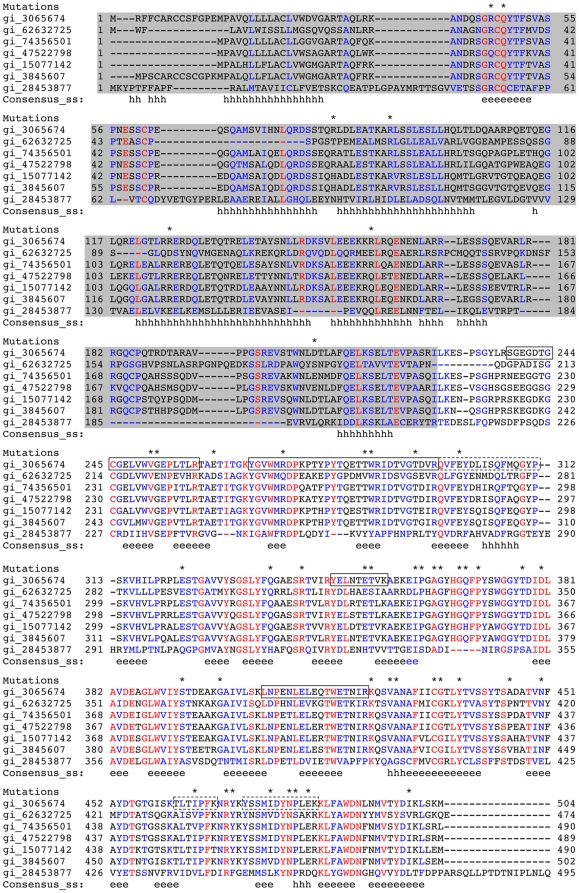
Multiple sequence alignment for myocilin and non-ocular ortholog amassin. Alignment includes myocilin from *H. sapiens* (gi 3065674), *D. rerio* (gi 62632725), *B. taurus* (gi 74356501), *S. scrofa* (gi 47522798), *M. musculus* (gi 15077142), *R. norvegicus* (gi 3845607), and amassin from *S. purpuratus* (gi 28453877). The region shaded in grey contains the N-terminal signal sequence and coiled-coil region, whereas the OLF domain remains unshaded. The consensus predicted secondary structures are shown in the last line (h, alpha-helix; e, beta-strand). Blue, similar residues; red, conserved residues; asterisk, reported disease-causing mutation in human myocilin [49]. The alignment and predicted secondary structure were generated using PROMALS [53]. Solid black boxes enclose peptides identified in both core-OLF and myoc-OLF by mass spectrometry; whereas dashed boxes enclose peptides identified only in myoc-OLF (see text).

Myocilin, the protein most closely associated with inherited forms of open angle glaucoma (OAG) through genetic linkage studies, is a ∼57 kDa glycoprotein composed of a secretion signal sequence, coiled-coil region, and a ∼30 kDa olfactomedin (OLF) domain (Figure 1), which harbors 90% of all reported pathogenic lesions [1]. The molecular mechanisms that lead to glaucoma are not well established but are of significant biomedical interest given that glaucoma is a leading cause of blindness worldwide, and early-onset myocilin glaucoma accounts for ∼4% of glaucoma cases, primarily afflicting children [1].

Wild-type (WT) myocilin is secreted from human trabecular meshwork (HTM) cells to the trabecular meshwork extracellular matrix (TEM) [2], [3], [4], the anatomical region believed to regulate intraocular pressure [4], [5]. By contrast, mutant myocilins aggregate in the endoplasmic reticulum (ER), leading to cell death and a malfunctioning matrix. The net result is an increase in intraocular pressure and retina degeneration, a hallmark of glaucoma [6]. A gain-of-toxic-function is thought to underlie the pathophysiology of myocilin glaucoma [7], [8]. Temperature-sensitive secretion of some myocilin variants [9], [10] indicates that when protein production is slowed, some mutant proteins appear native-like and competent for trafficking out of the cell and to the TEM. In cell culture, the toxicity of mutant myocilins can be reduced by the addition of certain chemical chaperones [11], [12], and in vitro, the compromised stability of mutant myoc-OLFs can be restored with some of the same compounds [13].

In spite of the importance of myocilin in inherited glaucoma pathogenesis, little is known about its normal biological function in the TEM, especially the OLF (myoc-OLF) domain. Full-length myocilin has been shown to bind to TEM proteins such as laminin and the Hep II domain of fibronectin [14], [15], as well as the glycosaminoglycan (GAG) heparan sulfate, but these interactions are localized to the coiled-coil region of myocilin, and not myoc-OLF [16]. The normal biological roles of myocilin are further complicated by reports of myocilin localized to the mitochondria of HTM cells [2], [17], calpain-II dependent cleavage prior to secretion [18], as well as its expression in other ocular tissues including sclera, ciliary body, iris, retina and optic nerve head [19]. Moreover, beyond the eye, OLF domains are found in numerous multicellular organisms, and more than half of reported OLF domains are found in neural tissues. OLF domains are proposed to play roles in neurogensis, neural crest formation, dorsal ventral patterning, cell-cell adhesion, cell-cycle regulation, cell-cell signaling, tumorigenesis, and have been implicated in psychiatric disorders [20]. The explicit roles of myocilin in any of these tissues and processes, however, are not clear due to a lack of functional assays.

The objective of this study is to probe the molecular properties of myoc-OLF to gain insight into its function and structure. The study was enabled by our recent development of a preparative in vitro expression system in which myoc-OLF is closely fused to a cleavable maltose binding protein (MBP) [13]. Our current work places the OLF domain for the first time in the context of other known proteins. The analysis of myoc-OLF described herein also provides a biophysical basis for the development of a new therapeutic avenue for glaucoma in the context of a protein conformational disorder.

## Results

### pH stability profile of myoc-OLF

We investigated the pH stability profile of myoc-OLF to elucidate any preferences of this domain as well as to gain insight into its resilience under different pH environments, such as low pH, that might be encountered under a known age-related TEM stressor, oxidative stress [21]. For the broad 96-well pH screen (Table S1), we used the MBP-OLF fusion protein and adapted the differential scanning fluorimetry (DSF) technique used originally to assess thermal stability differences between WT and glaucoma-causing myoc-OLF variants [13]. The melting of MBP is deconvoluted from the melting of OLF by the addition of 50 mM maltose. The melting curves of MBP and OLF are readily distinguished in the stability assay throughout the pH range tested. MBP is stable from pH 4-10.5 [22], OLF is not affected by the presence of maltose [13], and Sypro Orange is not pH sensitive in the range 4-10 (not shown).

To cross-validate the results obtained using the MBP-OLF fusion protein, we subsequently tested cleaved myoc-OLF in a subset of stabilizing and destabilizing buffers (Table S1). There was no difference in melting temperature (T_m_) between the MBP-OLF and cleaved myoc-OLF among the pHs tested (Table S1), reinforcing the independence of the domains in the fusion protein. Interestingly, myoc-OLF is stable in 100 mM buffers corresponding to a range in pH of 4.6 to 7.4, but specifically in sodium lactate, acetate, MES, phosphate and Hepes (Table 1, Table S1). Bicine at pH 7.0 and glycine at pH 8.2 were also stabilizing buffers, albeit within a more limited pH range (Table 1, Table S1). By contrast, OLF was destabilized in pH 4.0–4.6, as well as the full pH range tested of Bis-Tris, Imidazole, Tris, and CHES buffers (Table 1, Table S1).

**Table 1:** Summary of stabilization of myoc-OLF by buffers of varying pH.

Buffer	pH range	Stabilizing Buffer^a^
Sodium Lactate/HCl	4.0–4.4	No
**Sodium Lactate/HCl**	**4.8–5.2**	**Yes**
Sodium Acetate/Acetic Acid	4.2	No
**Sodium Acetate/Acetic Acid**	**4.6–6.2**	**Yes**
**MES/NaOH**	**5.0–7.4**	**Yes**
Bis-Tris/HCl	5.2–8.0	No
Imidazole/HCl	5.4–8.2	No
**K_2_HPO_4_/NaH_2_PO_4_**	**5.8–7.4**	**Yes**
K_2_HPO_4_/NaH_2_PO_4_	7.8–8.6	No
**Hepes/NaOH**	**6.0–7.2**	**Yes**
Hepes/NaOH	7.6–8.4	No
Tris/HCl	6.6–8.6	No
**Bicine/NaOH**	**7.0**	**Yes**
Bicine/NaOH	7.2–8.6	No
CHES/NaOH*	8.0–8.8	No
**Glycine/NaOH**	**8.2**	**Yes**
Glycine/NaOH	8.6	No

a No: T_m_<51.7°C; Yes: T_m_>51.7°C. The lower limit cutoff is 1°C less than the originally reported T_m_ of myoc-OLF in phosphate buffer, using DSF [13]. Full data are presented in Table S1.

The concentration dependence of buffer stabilization or destabilization was further investigated using acetate buffer pH 4.6 or Tris buffer pH 7.5 as examples, respectively (Table S1). Myoc-OLF is more stable in 10 mM Tris pH 7.5 compared to 100 mM Tris pH 7.5. For Tris buffer, increasing the ionic strength by the addition of NaCl partially restores stability, suggesting that counterions may play a role in this buffer system. Conversely, for acetate buffer pH 4.6, a further increase of 2°C in the T_m_ value for myoc-OLF is observed when 100 mM acetate buffer pH 4.6 is used compared to 10 mM (Table S1), with no additional effects due to the presence of NaCl (not shown).

### Stability analysis in the presence of GAGs

Interestingly, the preferred buffers for myoc-OLF contain sulfate and acetate functional groups that are reminiscent of GAGs, whereas those that are destabilizing in an overlapping pH range harbor aliphatic amines (Table 1). Thus, we sought to evaluate the stability of myoc-OLF in the presence of individual GAGs found in the TEM [23] at physiologically relevant concentrations [24] (Table 2). Indeed, a modest (ΔT_m_ = 4.5°C) stabilizing effect was observed for myoc-OLF in the presence of a relatively low concentration (0.75 mg/ml) of heparan sulfate, whereas a similar increase in stability for chondroitin sulfate and hyaluronic acid were observed only at higher concentrations of 10 mg/ml, at the outer limit of the physiological range, suggesting a weaker effect. The addition of dermatan sulfate did not change the T_m_ value of myoc-OLF in the full range tested.

**Table 2:** Stabilization of myoc-OLF by GAGs.

	T_m_ (°C)	ΔT_m_ (°C)
None	52.4±0.1	0
Chondroitin Sulfate	57.7±0.0	5.3
Dermatan Sulfate	52.7±0.0	0.3
Heparan Sulfate a	57.2±0.0	4.8
Hyaluronic Acid	55.4±0.1	3.0

a For these experiments, heparan sulfate concentration was 0.75 mg/ml, whereas all other GAGs were present at 10 mg/ml.

### Conformational analysis of myoc-OLF at pH 4.6, 5.8, and 7.2

In order to detect structural changes across the stable pH regime, we next compared the secondary structure of myoc-OLF by conducting CD melts in stabilizing buffers of varying pH. We measured the CD spectrum in 10 mM acetate buffer pH 4.6, below the calculated [25] pI = ∼5 of myoc-OLF, at the lower end of the stable range, as well as in 10 mM phosphate buffer pH 5.8 where OLF was the most stable in our assay (Table 1, Table S1), and at the physiological pH 7.2 expected in the TEM under normal conditions.

**Figure 2:**
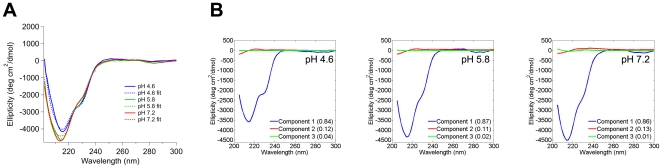
CD signatures of myoc-OLF domain at varying pH. (A) Prominent minima are observed at ∼217 nm and ∼230 nm for both experimental spectra (solid) and reconstructed spectral fit from SVD analysis (dashed). (B) Top component spectra from SVD analysis. Inset: relative contribution.

No major changes are observed among the three CD spectra at different pH values. The 200–210 nm range is more positive at pH 4.6 than the others, suggestive of non-native features of OLF in this buffer environment, but otherwise there are only slight differences in the intensity of the two major minima at ∼217 and ∼230 nm (Figure 2A). Component spectra, identified using singular value decomposition (SVD) analysis, reveal three significant spectral contributions. By far the most predominant component is one that contains both minima, followed by two minor contributions from spectra that resemble random coils (Figure 2B). Notably, there is no significant contribution from features at 208 nm and/or another at 222 nm; thus, there is no evidence of α-helices in myoc-OLF. The broad ∼217 nm minimum, which is characteristic of antiparallel β-sheets, resembles the CD spectrum of a smaller OLF construct expressed in *P. pastoris* [26], but the prominent shoulder near 230 nm has not been seen previously. The lack of this latter feature in the *P. pastoris* OLF construct may have been a result of its N-terminal truncation or the use of Tris pH 8.0 [26], a destabilizing buffer (Table 1, Table S1).

**Figure 3:**
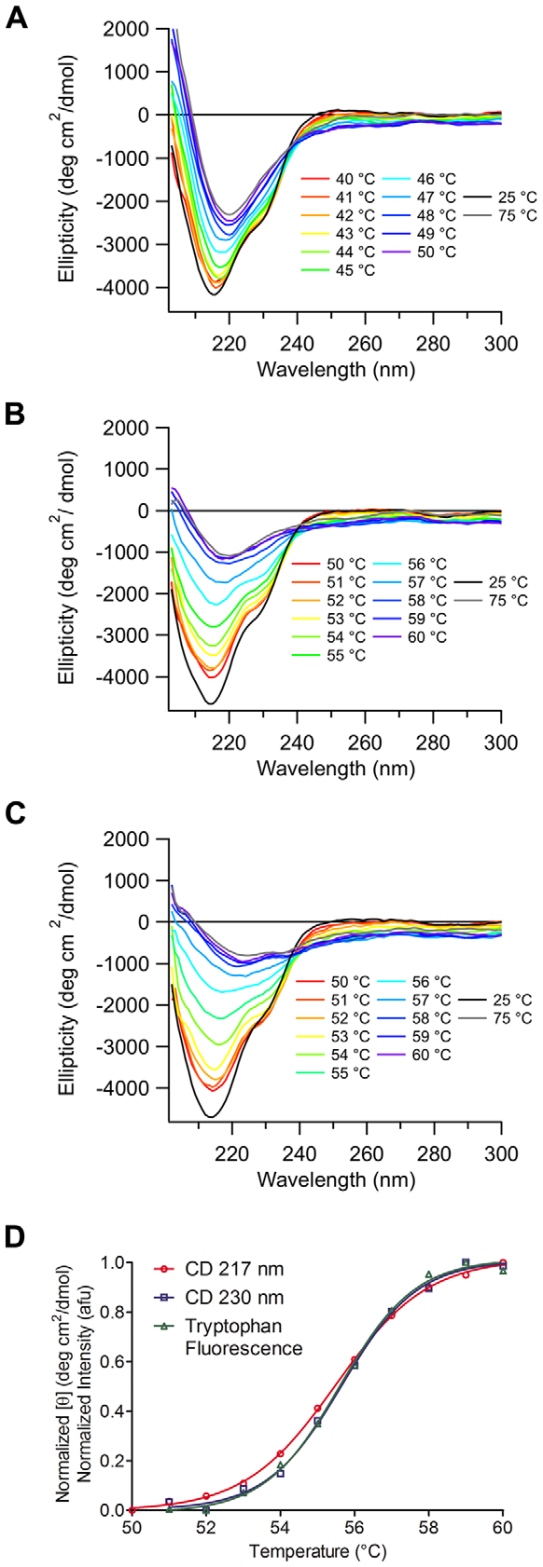
Thermal unfolding of myoc-OLF monitored by CD spectropolarimetry. Melts were conducted at (A) pH 4.6 (B) pH 5.8 and (C) pH 7.2. For each, 11 spectra are overlayed in the region of melting as well as two additional extrema. (D) Comparison of tryptophan fluorescence melt with CD melts from above. Each curve is normalized to the range of its respective signal.

Consistent with the aggregation propensity observed in mutants that lead to myocilin glaucoma, the unfolding transitions of myoc-OLF are irreversible. Sample precipitation was seen under all experimental conditions tested, including all concentrations, pH and temperature ranges. Nevertheless, there is substantial evidence for a highly cooperative transition [27]. First, at all three pH values, a sharp transition and similar isodichroic point near 238 nm are observed for myoc-OLF (Figure 3). Second, the differences in T_m_ for the melting curves monitored at the two local minima are consistently within 1°C of each other, and within error of the fit to the T_m_ obtained from the Boltzmann sigmoid equation, which assumes a two-state model (Table S2). Third, there is no obvious scan rate dependence of denaturation (not shown), suggesting that the melting transition is under thermodynamic, rather than kinetic control. Nevertheless, because we were not able to find experimental conditions of reversibility for myoc-OLF, the extent of the validity of thermodynamic parameters obtained using a two-state model cannot be assessed. We note, however, that thermodynamic parameters we obtained using this assumption with excellent error statistics (Text S1, Figure S1, Table S2) are similar to those of α-chymotrypsin [28], a protein that exhibits both similar mass and CD spectrum as myoc-OLF (see Discussion).

The transition to the denatured state for myoc-OLF at pH 4.6 occurs between 40–50°C (Figure 3A). Upon denaturation, the ellipticity is reduced by 50% and there is a shift in minimum at 216 nm in the folded state to 220 nm in the denatured state. SVD deconvolution (not shown) reveals a significant reduction in the β-sheet component and an increase in the random coil component, suggesting the precipitated sample is likely a molten globule with local secondary structure. By comparison, for both unfolding experiments conducted at pH 5.8 (Figure 3B) and pH 7.2 (Figure 3C), the transition occurs between 50 and 60°C with a fourfold reduction in signal intensity between folded and unfolded states of myoc-OLF. Less residual secondary structure remaining in these higher pH experiments may be attributed to the difference in net charge per residue. In conditions far from the pI of myoc-OLF, such as at pH 7.2, secondary structure would be expected to be disrupted due to the high net charge per residue. By contrast, at pH 4.6, myoc-OLF is closest to its pI where it may experience less repulsion and therefore retain secondary structure in the denatured state [29].

The T_m_ of myoc-OLF at pH 4.6 is nearly 10°C lower than the T_m_ at pH 5.8 or 7.2, and ∼3°C lower than that measured by DSF using the same ionic strength buffer (Table 1). Using a two-state assumption (see caveats above), comparison of the free energy of unfolding, as well as both enthalpy and entropy components, also indicate a lower barrier to unfolding (Table S2). Nevertheless, even the values at pH 4.6 are well within parameters obtained from numerous other folded proteins [28]. The thermodynamic values fit for myoc-OLF above its pI indicate a larger energy barrier and more stable protein, consistent with the measured increase in T_m_. Finally, the unfolding transition at neutral pH was further corroborated by a thermal melt in which intrinsic tryptophan fluorescence was monitored at pH 7.2 (Figure 3D). Indeed, the intrinsic T_m_ measured by both CD and Trp fluorescence melts is close to the T_m_ = 52.7°C we previously reported using the more facile, if indirect, DSF technique [13]. The melting thermogram overlays particularly well with the curve obtained monitoring ∼230 nm by CD, suggesting tryptophan residues contribute to this CD signal (see Discussion).

### Limits of the myoc-OLF core domain

We subjected myoc-OLF to limited proteolysis to identify its three dimensional core structure(s). In most constructs of myoc-OLF studied in the laboratory, a disulfide bond is formed between the only two available cysteine residues (Cys 245 and Cys 433) [13], [26], which are 189 residues apart. Yet, protein domains are typically composed of ∼150 amino acids [30]. It is possible that the two cysteine residues, far apart in sequence, are nevertheless topologically close. This could lead to either two or more smaller structural domains or one larger than average single domain.

**Figure 4:**
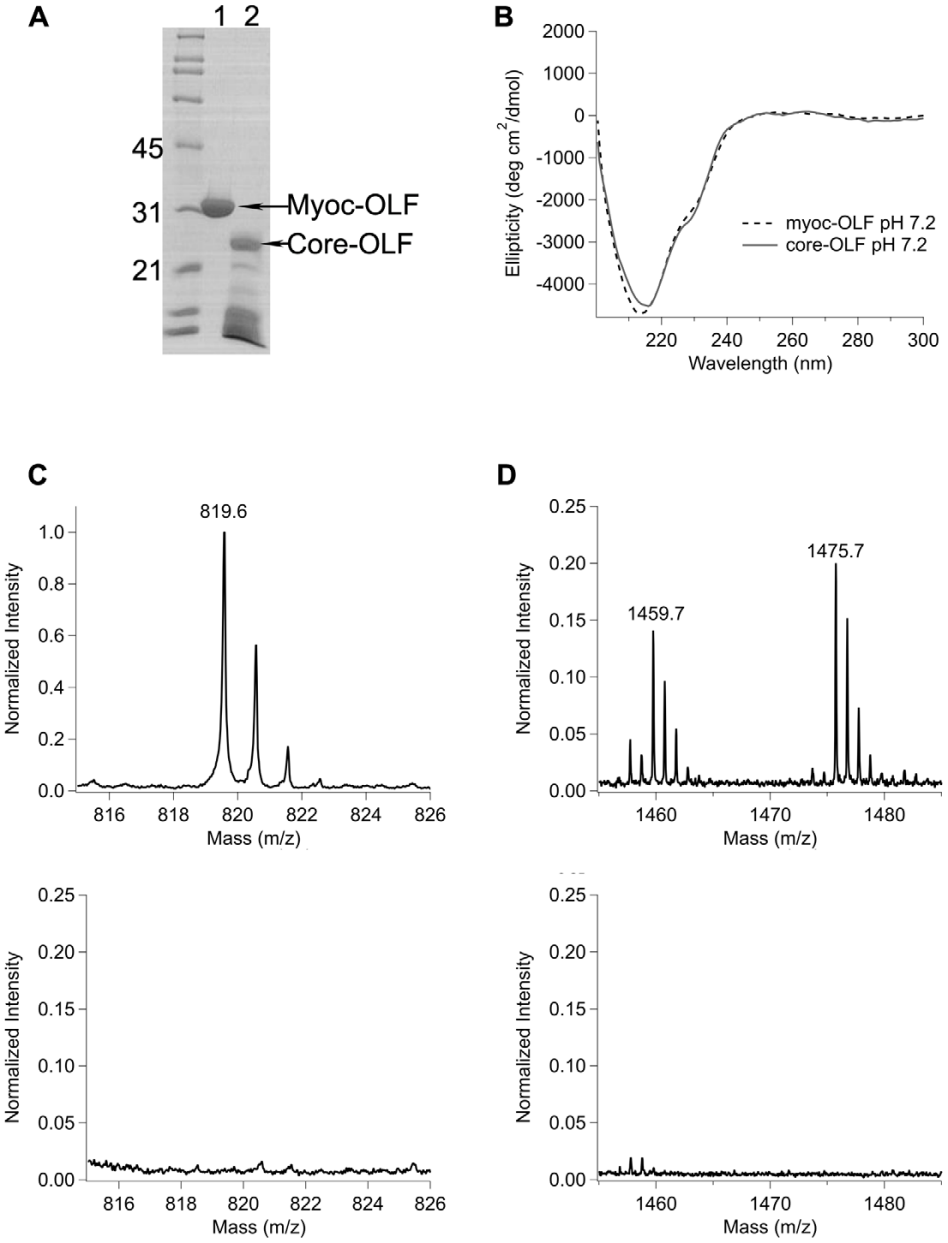
Analysis of core OLF domain. (A) SDS-PAGE analysis of myoc-OLF before and after limited proteolysis by subtilisin A. Lane 1, myoc-OLF, Lane 2, core-OLF. Molecular mass markers denoted in kDa. (B) Overlay of core-OLF and myoc-OLF CD spectra at pH 7.2. (C, D) Comparison of trypsin digest/MALDI TOF/TOF mass spectra of myoc-OLF (top) and core OLF domain (bottom) in two key regions (see text, Table 4).

Overall, myoc-OLF is resistant to cleavage by proteases at room temperature, including trypsin, α-chymotrypsin, pepsin, and V_8_ protease (not shown) suggesting that our construct consisting of residues 228–504 of myocilin, comprises a well folded, ∼30 kDa protein. However, incubation with subtilisin A, a non-specific protease that cleaves after large uncharged residues, generated a smaller domain of ∼25 kDa (Figure 4A). Similar results were obtained with the likewise promiscuous protease papain (not shown). The CD spectrum of core-OLF is similar to that of myoc-OLF with some minor exceptions, a 202 nm shoulder characteristic of a type I β-turn [31], and a more pronounced 230 nm shoulder (Figure 4B). As seen previously with myoc-OLF [13], the disulfide bond remains intact in core-OLF. Observed using the thiol-sensitive fluorogenic reagent, ThioGlo (see Materials and Methods), fluorescence increases only in samples in which the disulfide bond in core-OLF has been reduced with tris(2-carboxyethyl)phosphine (TCEP) (Table 3). Finally, the T_m_ is unchanged, 53.4±0.2°C measured by DSF.

**Table 3:** Characterization of disulfide bond in core-OLF.

Sample	Fluorescence Intensity (513 nm)
core-OLF (6 µM)	0.7
core-OLF (6 µM), TCEP (5 mM)	184.4

Peptide mass fingerprinting reveals that the N- and C-termini of core-OLF are truncated compared to myoc-OLF (Figure 1, Table 4). For core-OLF, the most N-terminal fragment observed in the spectrum encompasses residues 238–258, the extreme C-terminal peptide observed for core-OLF comprises residues 408–422 (Table 4), whereas the peptides TLTIPFK (residues 462–468, Figure 4C, Table 4) and YSSMIDYNPLEK (residues 473–484, Figure 4D, Table 4) are absent. The truncated C-terminal 44 residues may form a structural element not detected by SDS-PAGE due to its small size, or it may be largely unordered. In sum, consistent with the ∼25 kDa fragment size, and digest analysis, the main structural domain of myoc-OLF harbors a disulfide bond and is likely inclusive of residues Ser 238 and Lys 461.

**Table 4:** Observed and calculated mass spectrum peaks for myoc-OLF and core-OLF.

	Residue Range	Calculated Mass	Observed Mass	ΔMass	MS/MS	Sequence
Core-OLF	267–272	827.3868	827.3786	−0.0082		YGVWM^a^R
	288–296	975.5105	975.5100	−0.0005	yes	IDTVGTDVR
	347–355	1096.5521	1096.5477	−0.0044	yes	YELNTETVK
	273–287	1882.8970	1882.8944	−0.0026		DPKPTYPYTQETTWR
	406–422	2099.0403	2099.0400	−0.0003	yes	LNPENLELEQTWETNIR
	238–258	2232.0601	2232.0674	0.0073	yes	SGEGDTGC^b^GELVWVGEPLTLR
Myoc-OLF	462–468	819.4974	819.5486	0.0512		TLTIPFK
	267–272	843.3817	843.3954	0.0137		YGVWM^c^R
	288–296	975.5105	975.5311	0.0206		IDTVGTDVR
	347–355	1096.5521	1096.5712	0.0191		YELNTETVK
	473–484	1459.6672	1459.6852	0.0180		YSSMIDYNPLEK
	273–287	1882.8970	1882.9452	0.0482		DPKPTYPYTQETTWR
	406–422	2099.0403	2099.0972	0.0569		LNPENLELEQTWETNIR
	297–314	2180.0369	2180.0801	0.0432		QVFEYDLISQFMQGYPSK

a A indicates oxidation of methionine,

b B designates carbamidomethyl cysteine,

c C indicates methionine sulphone.

## Discussion

### Functional significance

The human TEM, a microenvironment in the eye consisting of fibrillar and curly collagens, elastic fibrils, basement membrane and amorphous basement membrane-like materials, as well as other specific proteins, proteoglycans and GAGs, is the anatomical region that controls outflow of aqueous humor in the eye [23]. The structural and functional details of this interconnected matrix, its receptor-mediated interactions with HTM cells, and mechanisms leading to phenotypes observed in the diseased state, are poorly understood [1]. The proper functioning of this complex tissue involves adaptation to a host of cellular and environmental stressors over time, and cumulative long-term detrimental effects of oxidative stress have been implicated in decreased aqueous humor outflow. Eventually, poor flow control leads to an impaired ability to regulate eye pressure, and subsequently, glaucoma [21]. Changes in GAG composition have also been observed in glaucoma-patient derived samples [32], [33], but the mechanisms that bring about these changes are unknown. In addition, the roles played by myocilin in the TEM, either normal or pathogenic, are not clear. In this study, we have unraveled some of the molecular characteristics of the myoc-OLF domain in the context of its TEM microenvironment, which will aid future functional characterization.

Several lines of evidence support the notion that GAGs provide a favorable environment for myoc-OLF to function, even though to date, no experiments have identified specific GAGs binders in the myoc-OLF region from any organism. Four GAG attachment sites to the myoc-OLF domain are predicted by the ELM database [34] within residues 232–235, 237–240, 330–333 and 443–446, suggesting that direct linkages are possible. The microenvironment of myoc-OLF in the TEM is also replete with GAGs at a total concentration of 1–2 mg/ml [35], with the approximate composition of 20–25% hyaluronic acid or hyaluronan, 40–60% chondroitin and dermatan sulfates, 5–10% karatan sulfate, and 15–20% heparan sulfate [23]. Our experiments reveal a modest increase in stability for myoc-OLF in the presence of heparan sulfate, chondroitin sulfate, and hyaluronic acid. Although no change in T_m_ was observed in the presence of dermatan sulfate, no GAGs tested destabilized myoc-OLF. Interestingly, whereas direct binding of chondroitin sulfate to another OLF-containing protein, mouse photomedin, has been shown [36], the presence of chondroitin sulfate only weakly influences thermal properties of myoc-OLF. This result suggests that chondroitin sulfate may have different functional significance for related OLF-containing proteins.

By contrast, a more pronounced effect on myoc-OLF stability was observed under physiologically relevant concentrations of heparan sulfate. Even though direct binding to the OLF domain has been ruled out by a previous study demonstrating binding via the N-terminal coiled-coil domain of myocilin [16], the knowledge that heparan sulfate binds to myocilin indicates that this GAG is in the local milieu of myoc-OLF. The observation that heparan sulfate thermally stabilizes myoc-OLF should prompt experiments to further clarify the nature of the mechanism of stabilization.

In addition, although heparan sulfate appears to exert a modest stabilizing effect on myoc-OLF in the absence of other analytes, it seems plausible that cations might enhance the favorable specific interactions between the negatively charged GAGs and the negatively charged OLF domain under physiological conditions. For example, detailed functional studies of amassin, a related, but non-ocular, OLF-containing protein (Figure 1) from the invertebrate animal sea urchin, demonstrates the requirement of the amassin OLF domain for cell-cell adhesion in coelomocytes. This process also requires the presence of Ca^2+^, and higher multimeric states of the amassin OLF domain [37]. To date, however, no canonical Ca^2+^-binding sites have been identified in myoc-OLF, and to the best of our knowledge, Ca^2+^ has not been included in any assays. In our hands, myoc-OLF does not bind Tb^3+^, a Ca^2+^ mimic [38] (not shown), and has only been isolated as a monomer by gel filtration [13]. Although it is possible that these two related proteins have unique modes of interactions and binding partners, based on our study we raise the possibility that certain key components, such as GAGs and/or metal ions, may be missing from functional assays of myocilin attempted to date.

Lastly, the importance of elucidating the mechanism by which GAGs stabilize myoc-OLF is underscored by the finding that the concentrations of GAGs in glaucomatous eyes deviate from those of healthy eyes [32], [33]. Specifically, concentrations of sulfated GAGs, such as chondroitin sulfate, are higher in samples derived from early-onset glaucoma patients than from controls [33], and the concentration of hyaluronan decreases in adult-onset OAG patient-derived specimens compared to controls [32]. Given the connection between myocilin and early-onset glaucoma [39], an understanding of the nuances of different GAG interactions with myoc-OLF may pave the way to a better comprehension of myocilin glaucoma pathogenesis.

### Structural significance

Although predominantly β-sheet-containing proteins, such as the immunoglobulins [40], exhibit a trough at ∼217 nm that is characteristic of antiparallel β-sheets [31], of particular interest is the prominent ∼230 nm shoulder that had not been observed previously. One possibility is that a β-turn [41] is present, a hypothesis supported by prediction of β-turns in myoc-OLF by bioinformatics [42]. Alternatively, or in addition, the 230 nm feature may arise from aromatic residues, as observed in certain serine proteases. For example, a notable 230 nm band in α-chymotrypsin has been attributed to a tryptophan residue whose conformation is sensitive to changes in its environment upon activation of α-chymotrypsinogen to α-chymotrypsin [43]. In support of this assignment, the Trp fluorescence melt curve overlays closely with that from 230 nm observed in CD (Figure 3D). Of the seven Trp residues in myoc-OLF, two, Trp 270 and Trp 286, are in and near, respectively, a proline-rich region. Alternatively, Trp 373 is within a region with significant disorder probability as predicted by GlobPlot [44]. Future studies involving systematic mutagenesis of each Trp residue may shed more light on the properties of this unusual feature of the CD spectrum.

Intriguingly, the CD spectrum of core-OLF is similar to that of the βγ-crystallin superfamily. Crystallins are cytoplasmic lens cell proteins associated with genetic forms of cataract, a condition in which the crystallins precipitate. Regardless of their quaternary structure, which often exhibits functionally significant polydisperse, domain-swapped oligomers [45], CD spectra of crystallins include the same features at ∼202 nm, ∼217 nm, and ∼230 nm as seen with core-OLF [46], albeit with different relative intensities. Myoc-OLF exhibits no significant sequence homology with crystallins, is ∼10 kDa larger, is not known to oligomerize, nor observed to harbor two structural domains [47]. Nevertheless, OLF likely shares structural features such as similar length or twist of its β-strands, and/or the unusually strained torsion angles in a β-hairpin. Further characterization of the three-dimensional structure of an OLF domain will help elucidate the extent of similarity of features with the crystallins, including repeat structures in myoc-OLF that may be important for myocilin self assembly and/or function in the TEM, or plausible molecular mechanisms for the severe aggregation properties observed upon thermal unfolding.

### Implications for new therapeutic directions

The details of the unfolding mechanisms of WT and disease-causing myoc-OLF domains variants, particularly at physiological pH, are informative for a pharmacological chaperone therapeutic effort [48] for myocilin glaucoma. In this approach, the binding of small molecules will enhance the stability of the mutant myocilins to WT levels so that after folding in the ER, mutant myocilins will meet quality control requirements and be competent for secretion to the TEM.

Both thermodynamics and kinetics may be important in chaperone therapy for myocilin glaucoma. The therapeutic small molecule might bind to a folded mutant protein to restore its stability to that of WT, and/or bind to a non-native conformation and accelerate folding to the native state. From this study we know that the ∼31 kDa WT myoc-OLF domain is a stable entity that appears to unfold under thermodynamic control in a highly cooperative transition. We can now move forward to compare unfolding pathways of the numerous missense mutants of OLF. If mutant OLFs unfold in similarly cooperative transitions, thermal stabilization of the fully folded protein may be sufficient for preventing aggregation and improving secretion, and screening for candidate chaperone molecules would be based on thermal stability alone. However, a more likely scenario is that deviations from two-state unfolding exist for at least some of the reported mutants [49]. In this case, a pharmacological chaperone may need to be tailored in such a way that it alters the folding pathway back to a fully cooperative mechanism observed in WT myoc-OLF. Future studies will examine to what extent such unfolding intermediates exist for specific glaucoma-causing myoc-OLF mutants.

## Materials and Methods

### Expression and purification of myoc-OLF

The myoc-OLF gene was introduced into the MBP fusion vector, pMAL-c4x, as described previously [13]. Expression and purification of MBP-OLF followed previously described procedures, as did generation of cleaved myoc-OLF by incubation with Factor Xa and further purification [13]. SDS-PAGE analysis was conducted as described [50].

### Thermal stability assay for buffer and pH analysis

Our fluorescence thermal stability assay [13] utilizing DSF [51] was adapted to identify buffer and pH preferences for myoc-OLF. Reactions of 30 µL were prepared at room temperature and delivered to 96-well optical plates (Applied Biosystems) before sealing with optical film (Applied Biosystems). The reaction mixture consisted of 1 µM myoc-OLF or MBP-OLF in 10 mM sodium phosphate dibasic/potassium phosphate monobasic, 200 mM NaCl pH 7.2 (Buffer A) and 5X Sypro Orange (Invitrogen). Buffers from pH 4 to pH 9 were obtained from the pHat™ Buffer screen (Emerald Biosciences) and added to each reaction at a final concentration of 100 mM, with the exception of N-Cyclohexyl-2-aminoethanesulfonic acid (CHES), which had a final concentration of 50 mM. Only for the initial screen was MBP-OLF used and 50 mM maltose added to the reaction mixture. Selected DSF measurements were repeated with cleaved myoc-OLF in acetate, MES, phosphate, Tris, bicine, and glycine buffers. Fluorescence data were acquired on an Applied Biosciences Step-One Plus RT-PCR instrument equipped with a fixed excitation wavelength (480 nm) and a ROX emission filter (610 nm). Melts were conducted from 25–95°C with a 1°C per min increase. Collected data were baseline subtracted, trimmed to include both the boundaries and the transition of interest, and subjected to Boltzmann sigmoid analysis (see below).

### Thermal stability assay in the presence of GAGs

The thermal stability assay described above was employed to test for increased myoc-OLF stability in the presence of GAGs including chondroitin sulfate (shark cartilage, Sigma), dermatan sulfate (TCI America), heparan sulfate (bovine kidney, Sigma), and hyaluronic acid (rooster comb, Sigma) at concentrations ranging from 0 mg/ml to 10 mg/ml. Samples were prepared in 10 mM Tris, pH 7.5 and 200 mM NaCl, diluted from a 5x stock solution. GAGs were added from 20 mg/ml stock solutions in water, keeping protein and Sypro Orange concentration the same as above. Data analysis was performed as described above.

### Circular dichroism (CD) spectropolarimetry

CD was performed on a Jasco J-810 spectropolarimeter with purified myoc-OLF (8–10 µM) prepared in Buffer A, Buffer A adjusted to pH 5.8, as well as in 10 mM sodium acetate/acetic acid and 200 mM NaCl, pH 4.6. Melts were performed in triplicate on each of the samples by raising the temperature from 5 to 90°C in 1°C /min increments using a Neslab RTE-111 (Thermo Scientific) circulating water bath and monitoring the profiles between 200 and 300 nm in a 0.1 cm cuvette. Ten spectra, scanned from 300 to 200 nm at rate of 500 nm•min^−1^, were then averaged at the designated temperature. Our attempts to acquire reliable data below 200 nm were not successful due to voltage limits of the instrument and available nitrogen flow rate. Temperature increase and spectra acquisition lasted about five minutes per degree Celsius, and no differences in melting transitions were observed when the scan rate was reduced to 0.5°C /min (data not shown). In all cases, samples precipitated after melting, including reversibility tests in which the temperature was raised just to the T_m_ and then cooled (not shown).

Each averaged spectrum was background-corrected and converted to mean residue ellipticity 
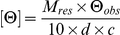
where *M_res_* = 112.9 is the mean residue mass calculated from the protein sequence; *θ*_obs_ is the observed ellipticity (degrees) at wavelength λ; *d* is the pathlength (cm); and *c* is the protein concentration (g/ml). CD melt data from 205 – 300 nm were deconvoluted into component spectra using the singular value decomposition procedure in Matlab (The Mathworks), and their statistical significance was calculated based on singular values. The top three components comprising ≥95% of the CD signal were then selected for reconstitution for the final fit plotted in Figure 2. Inspection of difference spectra confirmed that additional components consist of noise.

### Tryptophan fluorescence spectroscopy

Measurements were carried out in triplicate on a FluoroMax-3 spectrofluorimeter (Horiba Scientific). Intrinsic tryptophan fluorescence of myoc-OLF (2 µM) in Buffer A was excited at 280 nm (slit width 1 nm) and emission recorded in the range 300–400 nm (slit width 5 nm) with a maximum at 340 nm corresponding to tryptophan fluorescence emission. Each sample was heated from 20 to 70°C or 45 to 65°C, with a rate of three minutes per degree Celsius, using a Neslab RTE-7 Digital Plus (Thermo Scientific) circulating water bath.

### Boltzmann sigmoid analysis

The baseline-subtracted and trimmed melt curves acquired by the fluorescence thermal stability assay, CD, and intrinsic fluorescence were processed using GraphPad Prism. The reported T_m_ is the inflection point of the sigmoidal curve, and is calculated using the Boltzmann sigmoid equation 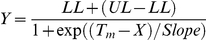



where *UL* and *LL* are the values of minimum and maximum intensities, respectively [51].

### Limited proteolysis

Myoc-OLF was pre-screened at room temperature against a variety of proteases including trypsin, α-chymotrypsin, pepsin, papain, V_8_ protease and subtilisin A to identify a protease and concentration capable of producing a discrete smaller construct detectable by SDS-PAGE. The optimal reaction condition consisted of a 1∶200 dilution of subtilisin A (Sigma Aldrich, 1 mg/ml) in 0.2 mg/ml myoc-OLF in 10 mM Hepes pH 7.5 or 1∶200 dilution of papain (Sigma, 1 mg/ml) in 0.2 mg/ml myoc-OLF in 10 mM MES pH 6.2. The reaction was incubated at room temperature for 30 minutes followed by subsequent quenching by either the addition of Complete Protease Inhibitor Cocktail (Roche) or SDS-PAGE sample loading buffer for SDS-PAGE analysis and in-gel digestion for mass spectrometry analysis. The core OLF product was fractionated from smaller digestion products on a Superdex 75 GL column (GE Healthcare) equilibrated with Buffer A. The core-OLF sample was further analyzed by CD as described above. Disulfide bond formation was confirmed with ThioGlo (EMD Biosciences) and the T_m_ measured as described [13].

### In-gel digestion and MALDI-TOF/TOF MS analysis

In-gel digestion of myoc-OLF and core-OLF was carried out as described previously [52]. Digested and dried samples were subjected to peptide mass fingerprinting analysis using the Georgia Institute of Technology Bioanalytical Mass Spectrometry Facility. Spectra were acquired on an Applied Biosystems 4700 Proteomics Analyzer MALDI-TOF/TOF tandem mass spectrometer. Core-OLF was also analyzed by MS/MS. Peaks were analyzed by using MASCOT (GPS Explorer, Applied Biosystems). Only identified peptide fragments with a>3∶1 signal-to-noise intensity were included in analysis. Samples were analyzed in duplicate. Due to the nonspecific nature of subtilisin cleavage, N-/C-terminal sequencing was not undertaken.

## Supporting Information

Figure S1 **Melt data fit to a two-state transition.** Melt curves and fitting residuals are presented. (A) pH 4.6, (B) pH 5.8, (C) pH 7.2. See values in Table S2.
(DOC)

Table S1 **Melting temperature of myoc-OLF and MBP-OLF in buffer.**
(DOC)

Table S2 **Thermodynamic data for myoc-OLF unfolding assuming a two-state transition.**
(DOC)

Text S1 **Supporting methods for two-state (Van't Hoff) thermodynamic analysis.**
(DOC)
