# Supplementary figures and images for: Biophysical Characterization of the Olfactomedin Domain of Myocilin, an Extracellular Matrix Protein Implicated in Inherited Forms of Glaucoma
Source: PLoS One. 2011 Jan 24;6(1):e16347. doi: 10.1371/journal.pone.0016347 (PMC3026022; doi:10.1371/journal.pone.0016347)

**Figure S1**


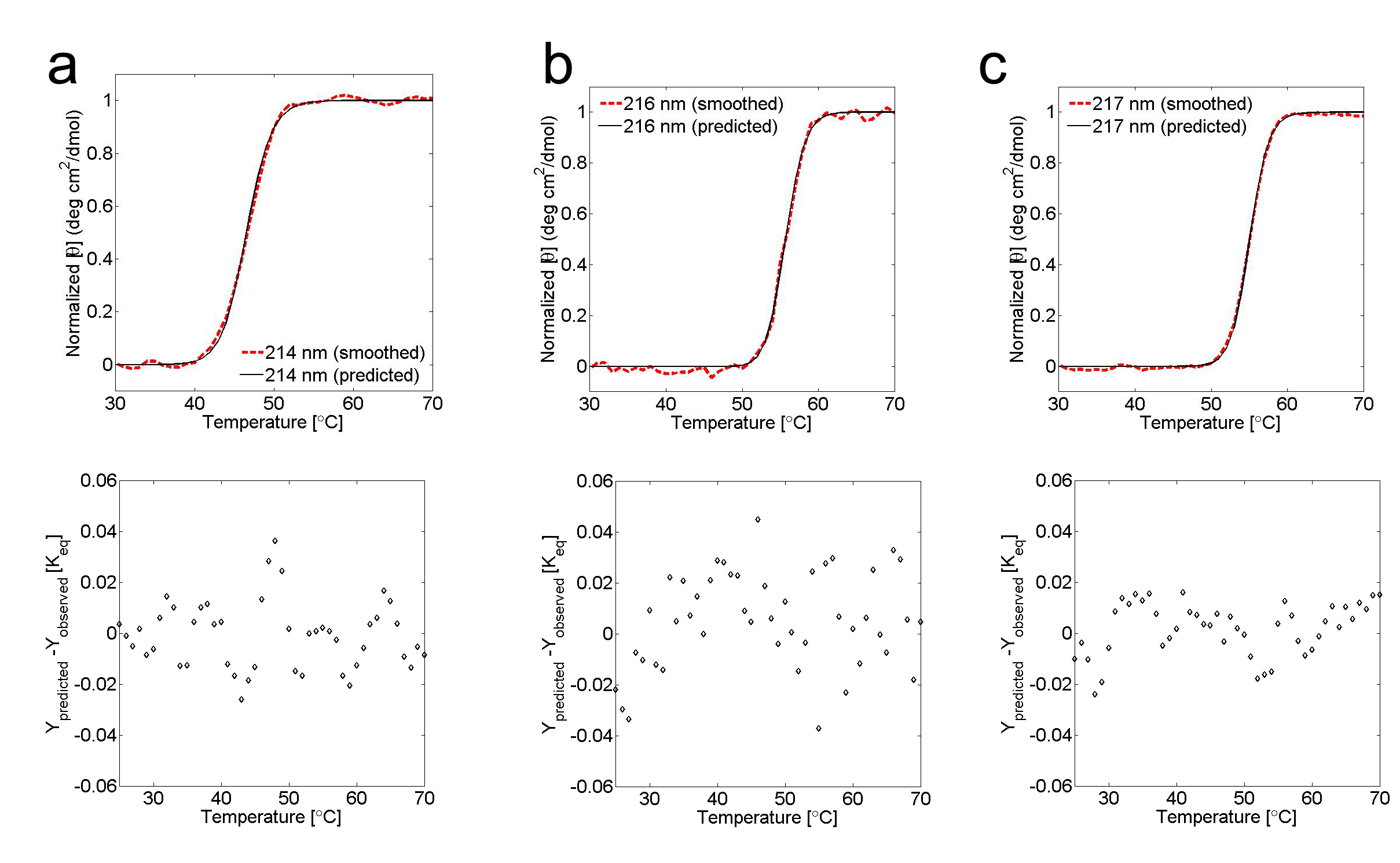

Supplement: Figure S1 — Melt data fit to a two-state transition. Melt curves and fitting residuals are presented. (A) pH 4.6, (B) pH 5.8, (C) pH 7.2. See values in Table S2. (DOC) [file pone.0016347.s001.doc]
