# Supplementary material for: Biophysical Characterization of the Olfactomedin Domain of Myocilin, an Extracellular Matrix Protein Implicated in Inherited Forms of Glaucoma
Source: PLoS One. 2011 Jan 24;6(1):e16347. doi: 10.1371/journal.pone.0016347 (PMC3026022; doi:10.1371/journal.pone.0016347)
Supplement: Table S1 — Melting temperature of myoc-OLF and MBP-OLF in buffer. (DOC) [file pone.0016347.s002.doc]

Table S1. Melting temperature of myoc-OLF and MBP-OLF in buffer.

| **Concentration** | **Buffer** | **pH** | **myoc-OLF *T_m_* (°C)** | **MBP-OLF *T_m_* (°C)** | **Δ *T_m_* (°C)**  **(myoc-OLF - MBP-OLF)** |
| --- | --- | --- | --- | --- | --- |
| 100 mM | Sodium Lactate/HCl | 4.0 |  | 39.4 ± 0.5 |  |
| 100 mM | Sodium Lactate/HCl | 4.4 |  | 47.1 ± 0.3 |  |
| 100 mM | Sodium Lactate/HCl | 4.8 |  | 52.2 ± 0.0 |  |
| 100 mM | Sodium Lactate/HCl | 5.2 |  | 55.4 ± 0.1 |  |
| 100 mM | Sodium Acetate/Acetic Acid | 4.2 | 47.7 ± 0.0 | 47.6 ± 0.5 | 0.1 |
| **10 mM** | **Sodium Acetate/Acetic Acid** | **4.6** | **49.8 ± 0.2** |  |  |
| 100 mM | Sodium Acetate/Acetic Acid | 4.6 | 51.9 ± 0.3 | 50.8 ± 0.2 | 1.0 |
| 100 mM | Sodium Acetate/Acetic Acid | 5.0 | 54.8 ± 0.3 | 54.0 ± 0.0 | 0.8 |
| 100 mM | Sodium Acetate/Acetic Acid | 5.4 | 56.5 ± 0.4 | 55.8 ± 0.1 | 0.7 |
| 100 mM | Sodium Acetate/Acetic Acid | 5.8 | 57.3 ± 0.1 | 56.2 ± 0.0 | 1.1 |
| 100 mM | Sodium Acetate/Acetic Acid | 6.2 | 56.6 ± 0.0 | 55.7 ± 0.2 | 0.9 |
| 100 mM | MES/ NaOH | 5.0 | 51.5 ± 0.2 | 51.3 ± 0.1 | 0.2 |
| 100 mM | MES/ NaOH | 5.4 | 53.8 ± 0.1 | 53.3 ± 0.1 | 0.5 |
| 100 mM | MES/ NaOH | 5.8 | 55.6 ± 0.1 | 54.7 ± 0.1 | 0.9 |
| 100 mM | MES/ NaOH | 6.2 | 55.8 ± 0.3 | 54.6 ± 0.0 | 1.2 |
| 100 mM | MES/ NaOH | 6.6 | 54.8 ± 0.2 | 53.9 ± 0.2 | 0.9 |
| 100 mM | MES/ NaOH | 7.0 | 53.2 ± 0.2 | 53.0 ± 0.1 | 0.3 |
| 100 mM | MES/ NaOH | 7.4 | 51.9 ± 0.0 | 52.1 ± 0.1 | -0.1 |
| 100 mM | Bis-Tris/HCl | 5.2 |  | 49.3 ± 0.1 |  |
| 100 mM | Bis-Tris/HCl | 5.6 |  | 50.8 ± 0.4 |  |
| 100 mM | Bis-Tris/HCl | 6.0 |  | 50.1 ± 0.1 |  |
| 100 mM | Bis-Tris/HCl | 6.4 |  | 48.6 ± 0.0 |  |
| 100 mM | Bis-Tris/HCl | 6.8 |  | 47.5 ± 0.2 |  |
| 100 mM | Bis-Tris/HCl | 7.2 |  | 46.7 ± 0.3 |  |
| 100 mM | Bis-Tris/HCl | 7.6 |  | 46.3 ± 0.1 |  |
| 100 mM | Bis-Tris/HCl | 8.0 |  | 46.2 ± 0.2 |  |
| 100 mM | Imidazole/HCl | 5.4 |  | 49.5 ± 0.4 |  |
| 100 mM | Imidazole/HCl | 5.8 |  | 49.4 ± 0.5 |  |
| 100 mM | Imidazole/HCl | 6.2 |  | 48.3 ± 0.5 |  |
| 100 mM | Imidazole/HCl | 6.6 |  | 47.0 ± 0.8 |  |
| 100 mM | Imidazole/HCl | 7.0 |  | 45.8 ± 0.2 |  |
| 100 mM | Imidazole/HCl | 7.4 |  | 45.4 ± 0.5 |  |
| 100 mM | Imidazole/HCl | 7.8 |  | 45.5 ± 0.6 |  |
| 100 mM | Imidazole/HCl | 8.2 |  | 45.9 ± 1.1 |  |
| **10 mM** | **K_2_HPO_4_/NaH_2_PO_4_** | **5.8** | **56.9 ± 0.2** |  |  |
| 100 mM | K_2_HPO_4_/NaH_2_PO_4_ | 5.8 | 56.1 ± 0.3 | 54.8 ± 0.0 | 1.2 |
| 100 mM | K_2_HPO_4_/NaH_2_PO_4_ | 6.2 | 55.2 ± 0.2 | 54.1 ± 0.1 | 1.0 |
| 100 mM | K_2_HPO_4_/NaH_2_PO_4_ | 6.6 | 54.3 ± 0.4 | 53.4 ± 0.1 | 0.9 |
| 100 mM | K_2_HPO_4_/NaH_2_PO_4_ | 7.0 | 51.8 ± 0.6 | 52.1 ± 0.3 | -0.3 |
| **10 mM** | **K_2_HPO_4_/NaH_2_PO_4_** | **7.2** | **55.3 ± 0.1** |  |  |
| 100 mM | K_2_HPO_4_/NaH_2_PO_4_ | 7.4 | 50.9 ± 0.9 | 51.0 ± 0.4 | -0.1 |
| 100 mM | K_2_HPO_4_/NaH_2_PO_4_ | 7.8 | 49.3 ± 0.3 | 49.9 ± 0.0 | -0.5 |
| 100 mM | K_2_HPO_4_/NaH_2_PO_4_ | 8.2 | 48.9 ± 0.0 | 49.4 ± 0.4 | -0.4 |
| 100 mM | K_2_HPO_4_/NaH_2_PO_4_ | 8.6 | 48.3 ± 0.5 | 48.9 ± 0.2 | -0.6 |
| 100 mM | Hepes/NaOH | 6.0 | 54.3 ± 0.1 | 53.4 ± 0.0 | 0.9 |
| 100 mM | Hepes/NaOH | 6.4 | 53.5 ± 0.0 | 52.7 ± 0.1 | 0.8 |
| 100 mM | Hepes/NaOH | 6.8 | 52.2 ± 0.1 | 52.3 ± 0.0 | -0.1 |
| 100 mM | Hepes/NaOH | 7.2 | 51.2 ± 0.3 | 51.1 ± 0.1 | 0.1 |
| 100 mM | Hepes/NaOH | 7.6 | 49.9 ± 0.3 | 50.4 ± 0.1 | -0.5 |
| 100 mM | Hepes/NaOH | 8.0 | 49.7 ± 0.3 | 49.7 ± 0.1 | 0.0 |
| 100 mM | Hepes/NaOH | 8.4 | 48.9 ± 0.2 | 49.2 ± 0.1 | -0.3 |
| 100 mM | Tris/HCl | 6.6 |  | 48.0 ± 0.0 |  |
| 100 mM | Tris/HCl | 7.0 |  | 46.3 ± 0.5 |  |
| 100 mM | Tris/HCl | 7.4 |  | 44.7 ± 0.3 |  |
| 100 mM | Tris/HCl | 7.5 | 44.5 ± 0.1 |  |  |
| 10 mM | Tris/HCl + 200 mM NaCl | 7.5 | 52.4 ± 0.1 |  |  |
| 100 mM | Tris/Cl + 200 mM NaCl | 7.5 | 50.9 ± 0.1 |  |  |
| 100 mM | Tris/HCl | 7.8 |  | 45.1 ± 1.6 |  |
| 100 mM | Tris/HCl | 8.2 |  | 43.5 ± 0.1 |  |
| 100 mM | Tris/HCl | 8.6 |  | 43.8 ± 0.1 |  |
| 100 mM | Bicine/NaOH | 7.0 | 51.9 ± 0.3 | 52.2 ± 0.0 | -0.2 |
| 100 mM | Bicine/NaOH | 7.4 | 50.0 ± 0.0 | 50.4 ± 0.6 | -0.4 |
| 100 mM | Bicine/NaOH | 7.8 | 48.5 ± 0.7 | 48.7 ± 0.1 | -0.2 |
| 100 mM | Bicine/NaOH | 8.2 | 47.5 ± 0.7 | 48.2 ± 0.2 | -0.7 |
| 100 mM | Bicine/NaOH | 8.6 | 47.2 ± 0.7 | 47.5 ± 0.3 | -0.4 |
| 50 mM | CHES/NaOH | 8.0 |  | 50.0 ± 0.7 |  |
| 50 mM | CHES/NaOH | 8.4 |  | 48.3 ± 0.3 |  |
| 50 mM | CHES/NaOH | 8.8 |  | 47.0 ± 0.3 |  |
| 100 mM | Glycine/NaOH | 8.2 | 51.3 ± 0.0 | 52.3 ± 0.6 | -1.0 |
| 100 mM | Glycine/NaOH | 8.6 | 47.9 ± 0.1 | 47.5 ± 0.1 | 0.3 |

*Bold indicates experiments used in conformation analysis (see main text)
