# Supplementary material for: Biophysical Characterization of the Olfactomedin Domain of Myocilin, an Extracellular Matrix Protein Implicated in Inherited Forms of Glaucoma
Source: PLoS One. 2011 Jan 24;6(1):e16347. doi: 10.1371/journal.pone.0016347 (PMC3026022; doi:10.1371/journal.pone.0016347)
Supplement: Table S2 — Thermodynamic data for myoc-OLF unfolding assuming a two-state transition. (DOC) [file pone.0016347.s003.doc]

**Table S2. Thermodynamic data for** myoc-OLF unfolding assuming a two-state transition.

|  | **Technique** | **Boltzmann Equation** | | **van’t Hoff Equation** | | | | | |
| --- | --- | --- | --- | --- | --- | --- | --- | --- | --- |
|  |  | *T_m_* (°C) | R^2^ | *T_m_* (°C) | Δ*G* (37 °C, kcal mol^-1^) | Δ*H_m_* (kcal mol^-1^) | Δ*S_m_* (cal mol^-1^K^-1^) | R^2^ | rmse |
| 4.6 | CD, 217 nm | 46.1 ± 0.1 | 0.997 ± 0.001 | 46.5 ± 0.0 | 3.5 ± 0.4 | 118.4 ± 13.3 | 370.5 ± 41.7 | 0.954 ± 0.037 | 0.000 ± 0.001 |
|  | CD, 230 nm | 46.9 ± 0.2 | 0.997 ± 0.003 | 47.0 ± 0.7 | 3.8 ± 0.6 | 120.9 ± 23.9 | 378.0 ± 75.0 | 0.997 ± 0.001 | 0.014 ± 0.007 |
| 5.8 | CD, 216 nm | 55.3 ± 0.1 | 0.997 ± 0.000 | 55.5 ± 0.0 | 9.0 ± 0.6 | 158.1 ± 11.0 | 481.0 ± 33.9 | 0.920 ± 0.015 | 0.001 ± 0.001 |
|  | CD, 230 nm | 56.1 ± 0.1 | 0.997 ± 0.001 | 56.5 ± 0.0 | 9.7 ± 0.4 | 156.0 ± 8.0 | 474.0 ± 24.0 | 0.913 ± 0.017 | 0.001 ± 0.013 |
| 7.2 | CD, 214 nm | 55.2 ± 0.2 | 0.998 ± 0.000 | 55.5 ± 0.0 | 8.85 ± 1.1 | 159 ± 22.0 | 484.0 ± 67.9 | 0.953 ± 0.014 | 0.000 ± 0.002 |
|  | CD, 229 nm | 55.8 ± 0.4 | 0.997 ± 0.004 | 56.0 ± 0.7 | 8.4 ± 0.2 | 146.4 ± 9.5 | 445.0 ± 29.7 | 0.903 ± 0.011 | 0.009 ± 0.005 |
|  | Fluorescence Stability Assay | 52.7 ± 0.8 | 0.998 ± 0.001 | N/A | N/A | N/A | N/A | N/A | N/A |
|  | Tryptophan Fluorescence | 55.6 ± 0.1 | 0.998 ± 0.001 | 55.0 ± 0.0 | 10.4 ± 0.1 | 180.5 ± 3.4 | 548.5 ± 10.6 | 0.991 ± 0.009 | 0.015 ± 0.005 |
