# Supplementary material for: Biophysical Characterization of the Olfactomedin Domain of Myocilin, an Extracellular Matrix Protein Implicated in Inherited Forms of Glaucoma
Source: PLoS One. 2011 Jan 24;6(1):e16347. doi: 10.1371/journal.pone.0016347 (PMC3026022; doi:10.1371/journal.pone.0016347)
Supplement: Text S1 — Supporting methods for two-state (Van't Hoff) thermodynamic analysis. (DOC) [file pone.0016347.s004.doc]

**Text S1.** **Supporting methods for two-state (Van’t Hoff) thermodynamic analysis.**

*Van’t Hoff analysis*- Thermal denaturation curves from CD and fluorescence melts were analyzed assuming a two-state model. The data were normalized and the protein fraction present in the unfolded conformation (*f_U_*), equilibrium constant (*K_eq_*), and Gibbs free energy (Δ*G*) were calculated using the following equations:

(1)

(2)

(3)

where U and F represent the concentration of myoc-OLF in the unfolded and folded states, respectively, R is the gas constant, and T is the absolute temperature (K) [1]. The data were analyzed by a nonlinear least-squares fit (eq 4) taking into account the v’ant Hoff analysis (eq 5):

$\frac{Y=(yF+mFT)+(yU+mUT)\exp[(\Delta S_{m}/R)-(\Delta H_{m}/RT)]}{1+\exp[(\Delta S_{m}/R)-(\Delta H_{m}/RT)]}$ (4)

$\ln K_{eq}=(-\Delta H_{m}/R)(1/T)+(\Delta S_{m}/R)$ (5)

where *Y* is the measured ellipticity. The pre-transition baseline slope and intercept are described by mF and *yF*, respectively, while *mU* and *yU* are the slope and intercept of the post-transition baseline. Furthermore, Δ*H_m_* is the v’ant Hoff enthalpy and is the slope of the plot of ln K_eq_ versus 1/T, (-Δ*H_m_*/*R*), and Δ*S_m_* is y-intercept (Δ*S_m_*/*R*) [2,3]. The residual (R^2^) represents the error in linearity of the calculated v’ant Hoff plot, whereas root mean square error (rmse) calculates the error between the smoothed observed and predicted models (see Table S2).

**References**

1. Greenfield NJ (2006) Using circular dichroism collected as a function of temperature to determine the thermodynamics of protein unfolding and binding interactions. Nat Protoc 1: 2527-2535.

2. Santoro MM, Bolen DW (1988) Unfolding free-energy changes determined by the linear extrapolation methods. 1. Unfolding of phenylmethanesulfonyl alpha-chymotrypsin using different denaturants. Biochemistry 27: 8063-8068.

3. Freire E (1995) Thermal denaturation methods in the study of protein folding. Energetics of Biological Macromolecules. San Diego: Academic Press Inc. pp. 144-168.
